# Excellent accuracy of ABC/2 volume formula compared to computer-assisted volumetric analysis of subdural hematomas

**DOI:** 10.1371/journal.pone.0199809

**Published:** 2018-06-26

**Authors:** Sae-Yeon Won, Andrea Zagorcic, Daniel Dubinski, Johanna Quick-Weller, Eva Herrmann, Volker Seifert, Juergen Konczalla

**Affiliations:** 1 Department of Neurosurgery, University Hospital, Goethe-University, Frankfurt am Main, Germany; 2 Deparment of Neuroradiology, University Hospital, Goethe-University, Frankfurt am Main, Germany; 3 Institute of Biostatistics and Mathematical Modelling, Department of Medicine, Frankfurt am Main, Germany; George Washington University, UNITED STATES

## Abstract

**Background:**

Subdural hematoma (SDH) is a common disease associated with high morbidity, which is becoming more prominent due to the increasing incidence. Decision for a surgical evacuation is made depending on the clinical appearance and the volume of SDH, wherefore it is important to have a simple ‘bedside’ method to measure and compare the volume of SDH.

**Objective:**

The aim of the study was to verify the accuracy of the simplified ABC/2 volumetric formula to determine a valuable tool for the clinical practice.

**Methods:**

Preoperative CT-scans of 83 patients with SDHs were used for the computer-assisted volumetric measurement via BrainLab® as well as the ABC/2 volumetric measurement. A = largest length (anterior to posterior) of the SDH; B = maximum width (lateral to midline) 90° to A; C = maximum height (coronal plane or multiplication of slices) of the hematoma. These measurements were performed by two independent clinicians in a blinded fashion. Both volumes were compared by linear regression analysis of Pearson and Bland-Altman regression analysis.

**Results:**

Among 100 SDHs, 53% were under an 47% were over 100cm^3^ showing a well distribution of the hematoma sizes. There was an excellent correlation between computer-assisted volumetric measurement and ABC/2 (R^2^ = 0.947, p<0.0001) and no undesirable deviation and trend were detected (p = 0.101; p = 0.777). A 95% tolerance region of the ratios of both methods was [0.805–1.201].

**Conclusion:**

The ABC/2 method is a simple and fast bedside formula for the measurement of SDH volume in a timely manner without limited access through simple adaption, which may replace the computer-assisted volumetric measurement in the clinical and research area. Reason for the good accuracy seems to be the spherical form of SDH, which has a similarity to a half ellipsoid.

## Introduction

Subdural hematoma (SDH) is a common disease and is becoming more prominent due to increasing incidence as well as cost factor national wide [1,2]. The decision for a surgical evacuation is made by the clinical appearance, mass effect with brain herniation and the volume of SDH, wherefore it is important to have a simple bedside method to measure the volume of SDH in clinical routine. Furthermore, an easy and valid method of hematoma volume is essential to make studies comparable. Previously, several studies investigated and reported the accuracy of the simplified ellipsoid volumetric formula, ABC/2, to measure intracerebral hemorrhage (ICH) as well as epidural hematoma (EDH), whereas to the best of our knowledge, there have been only one study observing the formula regarding SDH in small number of patients [3–7]. Since the geometric form of SDH differs from an ICH or EDH, which are ellipsoidal, we hypothesized an inaccuracy of ABC/2 for the volumetric measurement of SDH. Therefore, the aim of the study was to compare the ABC/2 formula to a computer-assisted volumetric analysis and in case of an inaccuracy to create a new formula for SDH volume measurement.

## Subjects and methods

This study was approved by the clinical ethic committee of the university Frankfurt (EK Nr.509/15). The ethic committee waived the need for patient content. In this study, we analysed 82 patients with chronic SDH from 2016 to 2017. Both sided SDH was accounted as two SDHs resulting in a review of 100 subdural hematomas. The preoperative CT-scans at admission were performed in a 5mm slice thickness and were used for both volumetric measurements: the computer-assisted measurement and ABC/2. Each method was used by an independent clinician in a blinded fashion. Patients with combined interhemispheric SDH or missing radiological data were excluded.

**Fig 1:**
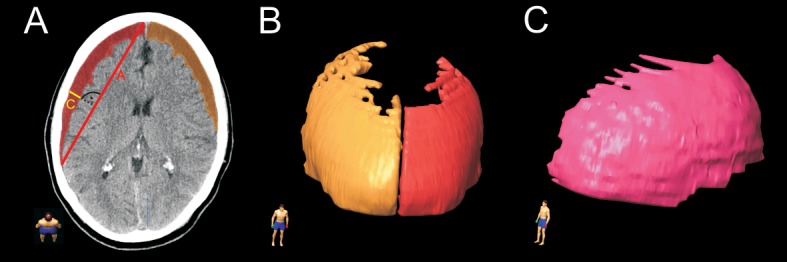
**A.** Preoperative axial CT-scan with both sided subdural hematomas (SDH). Computer-assisted volumetric measurement was performed by hand tracing each hematoma form in each slice as shown in red and orange colour. ABC/2 measurement technique was performed as following: A is the length connected each corner of the SDH, B is 90° to A and C is the height calculated by the slice thickness with visible hematoma. **B.** 3D frontal reconstruction of computer-assisted volumetric measurement of both sided SDH. **C.** 3D lateral reconstruction of computer-assisted volumetric measurement of single sided SDH.

For the ABC/2 method axial CT planes were used. The largest length (anterior to posterior) to each corner of the SDH was defined as A (cm), the maximum width (lateral to midline) 90° to A in the same slice as B (cm) and the maximum height of the hematoma as C (cm). For the calculation of the height, the number of slices with visible hematoma was multiplied by the thickness (e.g. 5mm) of the CT-scan or a coronal plane was used. The hematoma volume (cm^3^) was obtained by multiplying A, B and C and dividing it by 2 (Fig 1A).

For the computer-assisted measurement, the BrainLab® elements software (Brainlab Germany Headquarters, Munich, Germany) was used. This software can be used for radiosurgical therapy planning, intraoperative navigation and preoperative segmentation including multiplanar volume definition. The hematoma margins were hand traced and the volume was automatically calculated (Fig 1B and 1C).

Linear regression analysis of Pearson and Bland-Altman regression analysis on log-scale were used to determine the correlation between those methods [8].

## Results

**Fig 2:**
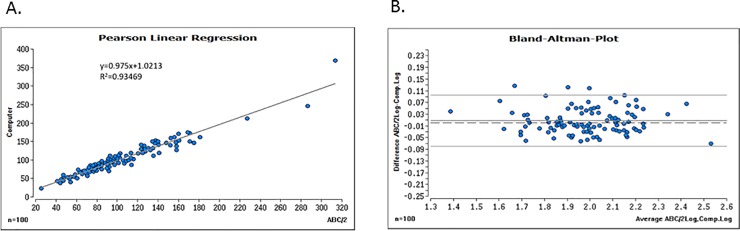
**A.** Pearson Linear regression analysis between ABC/2 and computer-assisted volumetric measurement. **B.** Bland-Altman regression analysis between log of ABC/2 and log of computer-assisted volumetric measurement.

Among 100 SDHs, 53 SDHs (53%) were under and 47 SDHs (47%) were over 100cm^3^ showing well distributed hematoma size. The mean volume was 106.3±47.3cm^3^ in the ABC/2 technique whereas 104.6±47.7cm^3^ in the computer-assisted volumetric measurement showing no significant difference. In the linear regression analysis, there was a highly significant correlation between ABC/2 and computer-assisted values (R^2^ = 0.934, slope = 0.975) (Fig 2A). The same result was also observed in the Bland-Altman regression analysis between the log of ABC/2 and the log of the computer-assisted values (R^2^ = 0.947, p<0.0001) (Fig 2B). Bland and Altmann regression revealed no undesirable deviation between the geometric mean of ABC/2 and the computer-assisted measurements and the ratios of these values (p = 0.101) and no undesirable trend (p = 0.777). A 95% tolerance region of the ratios of both methods was [0.805–1.201].

## Discussion

Against our initial hypothesis there was an excellent correlation between ABC/2 formula and computer-assisted volumetric measurement of SDH supporting the use of ABC/2 in the clinical practice and research. In addition, the formula had no limited access to the size of SDH volume.

At this point, the relevance of this simplified ABC/2 formula in patients with SDH should be mentioned. Previously Kothari et al. analyzed the timely manner of each volumetric measurement indicating that the simplified formula required less than one minute, whereas the computer-assisted volumetric analysis required about 15 factors more time [4]. There is a well-known phrase in the treatment of stroke, “time is brain”. However, the time is also essential in case of any traumatic brain injuries like SDH since an accurate initiation of acute management can reduce morbidity and improve further prognosis [2,9]. Additionally, the formula can be used as a helpful tool for the further evaluation of SDH residuum at follow-up and further studies to compare each volumes without any specific software. In certain circumstances, it is not simple to compare the development of SDH volume due to the change of geometric SDH form, varying thickness of scans or slice variations. Indeed measuring only the maximum thickness of the hematoma might illustrate us false information which could result in an unnecessary surgical treatment. Therefore, by using the simplified formula, the comparison could be made promptly and ease the further decision.

**Fig 3:**
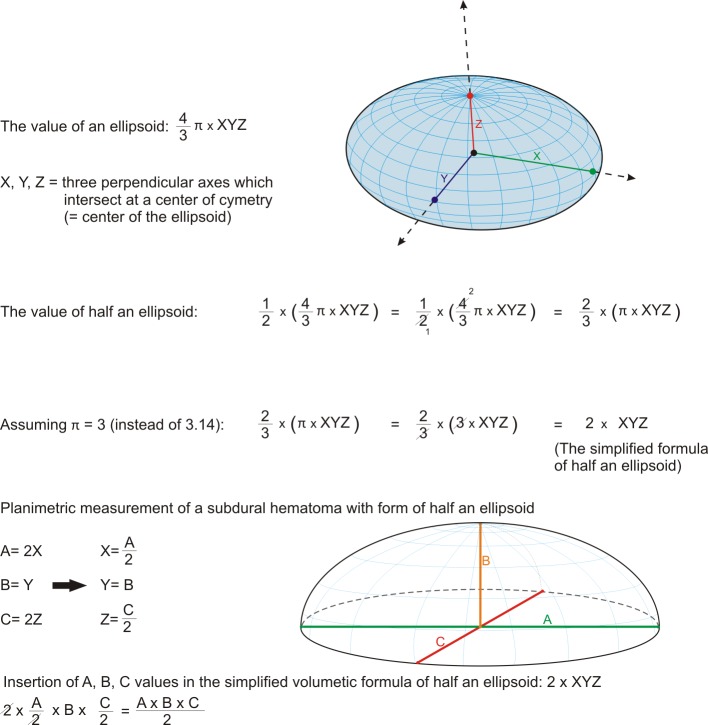
Mathematical derivation of the ABC/2 formula from a half ellipsoid volumetric formula.

For the volumetric measurement of ICH, several studies have been estimating using different type of formula, however, only the formula for an ellipsoid have been shown to correlate well with plan metric techniques [4,10–12]. In addition, this formula was approved for several other entities like EDH, vestibular schwannomas and gliomas, whereas for cerebral arteriovenous malformation a significant discrepancy was identified, possibly due to the heterogeneous group of lesions [7,13–15]. To date, there is only one study published, GUSTO-1 trial, showing the accuracy of ABC/2 in the volumetric measurement of SDH similar to our study [4]. However in this study, the mean volume of the SDH (68cm^3^) and the sample size (n = 40) were small. Since there could be a discrepancy concerning the volume, we included well distributed size of the SDH (range 57–363 cm^3^), which showed no limited use of this formula. Furthermore by increasing the sample size, we tried to obtain more statistical power in order to replace the computer-assisted volumetric measurement,

Against our initial expectation we questioned, why the volume of SDH would fit into this formula for an ellipsoid: The simple explanation for this might be the spherical form of SDH, which has a similarity to a half ellipsoid. Detailed information is described below in Fig 3.

There are some limitations to mention in this study. We excluded SDH with interhemispheric components, since those geometric forms are completely different than lateral SDH. There might be other formula to add those components; however in this study we just focused on the lateral component. Secondly, we used CT-scans with 5mm thickness slices. The calculation of the hematoma size could be adjusted depending on the slice thickness, but there might be some discrepancy using CT-scans with thicker slices.

## Conclusion

In this study, we identified that the ABC/2 method is a simple and excellent bedside formula, which can be used for the volume measurement of SDH without limited access in order to initiate acute management and decision in a timely manner. Reason for the good accuracy seems to be the spherical form of SDH, which has a similarity to a half ellipsoid.
